# A new fuzzy method for investigating the effects of dam on aquifer: case study of Rudbal dam, south of Iran

**DOI:** 10.1038/s41598-024-65353-1

**Published:** 2024-06-24

**Authors:** Sajjad Moradi Nazarpoor, Mohsen Rezaei, Fateme Mali

**Affiliations:** Faculty of Earth Sciences, Shiraz University, Shiraz, Iran

**Keywords:** Mann–Kendall, Fuzzy method, Groundwater, Rodbal dam, Environmental sciences, Hydrology

## Abstract

Dam construction has some qualitative and quantitative effects on groundwater resources. This effect may be in the form of an increasing groundwater table or a changing groundwater system. In this paper, the effect of the Rodbal dam construction on the Darab aquifer was investigated. For this paper water table levels from observation wells and precipitation data was used. The study aims to analyse the behaviour of the water table during precipitation by employing a combination of the standardisation process, Fuzzy Inference System (FIS), and Root Mean Square Error (RMSE) calculations. Fuzzy logic involves the fuzzification of input data, transforming precise values into fuzzy sets. The effectiveness of the FIS is highlighted, particularly in determining the number of membership functions for inputs. The performance of the results is assessed using indicators such as RMSE and Coefficient of Determination (R2). The FIS showed a high level of effectiveness in performance assessment, exhibiting a 70% similarity between the fuzzy and Mann–Kendall methods. Nonetheless, the Fuzzy Purpose Method corresponded more closely with the observed data, indicating a more accurate reflection of reality. The findings indicate that for P3, P7, and P8, the results from the Mann–Kendall method do not show a discernible trend. Conversely, the proposed Fuzzy method accounts for changes in the behaviour of these piezometers.

## Introduction

It is evident that dam construction can always affect groundwater. However, any defect in the dam structure can have an influence on groundwater fluctuations, which is particularly important in semi-arid and arid regions. The role of dams in utilizing surface water and fostering human development is crucial. Therefore, ensuring the maintenance of the dam structure is of utmost importance for the management group. If the dam structure is not executed flawlessly, the goals of the plan will not be achieved and capital will be wasted.

Dams have the potential to impact hydrologic regimes because of their ability to store a significant volume of water. Despite the difficulties, recent years have witnessed the successful development of methods that can investigate the impact of the dam construction such as dams, climate changes, earthquakes, and other natural phenomena. Time-series analysis is one method that can identify water quality and quantity trends simultaneously . Thus, other techniques such as statistical methods are being currently used to investigate analytical time trend and survey the patterns of the time series trend^1–7^.

On the one hand, there are non-parametric and parametric methods to investigate the trends of a study’s time series, but on the other hand, the non-parametric is preferred for use^8^. However, non-parametric methods are often preferred when assumptions for applying parametric methods such as stationarity and independency of time series are not met. This is because the assumptions for using parametric methods are often too limited. The non-parametric methods are more practical, making them a better choice when parametric assumptions are not met.Ultimately, non-parametric methods are often the preferred choice in situations where parametric assumptions are not met. Additionally, parametric methods are sensitive to outlier data, meaning that one extreme point or value can have a disproportionately large effect on the results. Non-parametric methods, on the other hand, are not as sensitive to outliers data^9^.

Non-parametric methods which can be applied to investigate time series analysis, like Long-Term Persistence (LTP), Trend-Free Pre-Whitening (TFPW), Shorahmadi2018spatiotemporalt-Term Persistence (STP), and Mann–Kendall^10–13^. Among non-parametric methods, Mann–Kendall is one that stands out as the most frequently used. To use the Mann–Kendall test, lack of autocorrelation is essential and the only condition for time series data. This is because the presence of correlation between time series data can lead to varying results; without autocorrelation, the Mann–Kendall test will be able to provide accurate results^10,11^. In general, researchers are always interested in investigating the influence of dams over time.

In addition, this study contends that the fuzzy method, based on the existence of the new method, can be used to determine the influence of dam structures. Fuzzy logic is a key approach that is widely applied in various scientific, such as investigating of groundwater quality^14^, harnessing expert knowledge to great effect^15^. Therefore, fuzzy logic decreases the uncertainty deals by the collection of expert knowledge^16^. novelty of this research lies in its application of fuzzy logic to investigate the effects of dam construction on groundwater, a method not commonly applied in previous studies. Traditional methods, such as the Mann–Kendall test, rely on non-parametric statistics, which may not fully capture the complexity of interactions between dam operations, precipitation. To draw important conclusions, it is necessary to conduct a thorough examination of fuzzy logic. fuzzy inference systems consist of four steps: Fuzzyification, fuzzy rule base, fuzzy inference and denazification. Fuzzification is the step that converts precise inputs into the fuzzy set. The relation between fuzzy sets is defined by the fuzzy rules base. Fuzzy inference processes apply the fuzzy set through the fuzzy rules base to make inferences. The process of transforming indistinct outcomes into clear and precise outputs is denazification.

## Area of study

The Darab plain located in the southeastern part of Shiraz province. This remarkable area is situated at a latitude of 20 and a longitude of 50. The elevation in this plain has an average of 2000 m, and Darab Aquifer as study area is covers a total of 693 km. The average annual precipitation and temperature are 245 mm and 22 $^{\circ }$C, respectively.The Rodbal River is the most important river in the Darab Plain, which is located along the rodbal-Stahban road.

**Figure 1:**
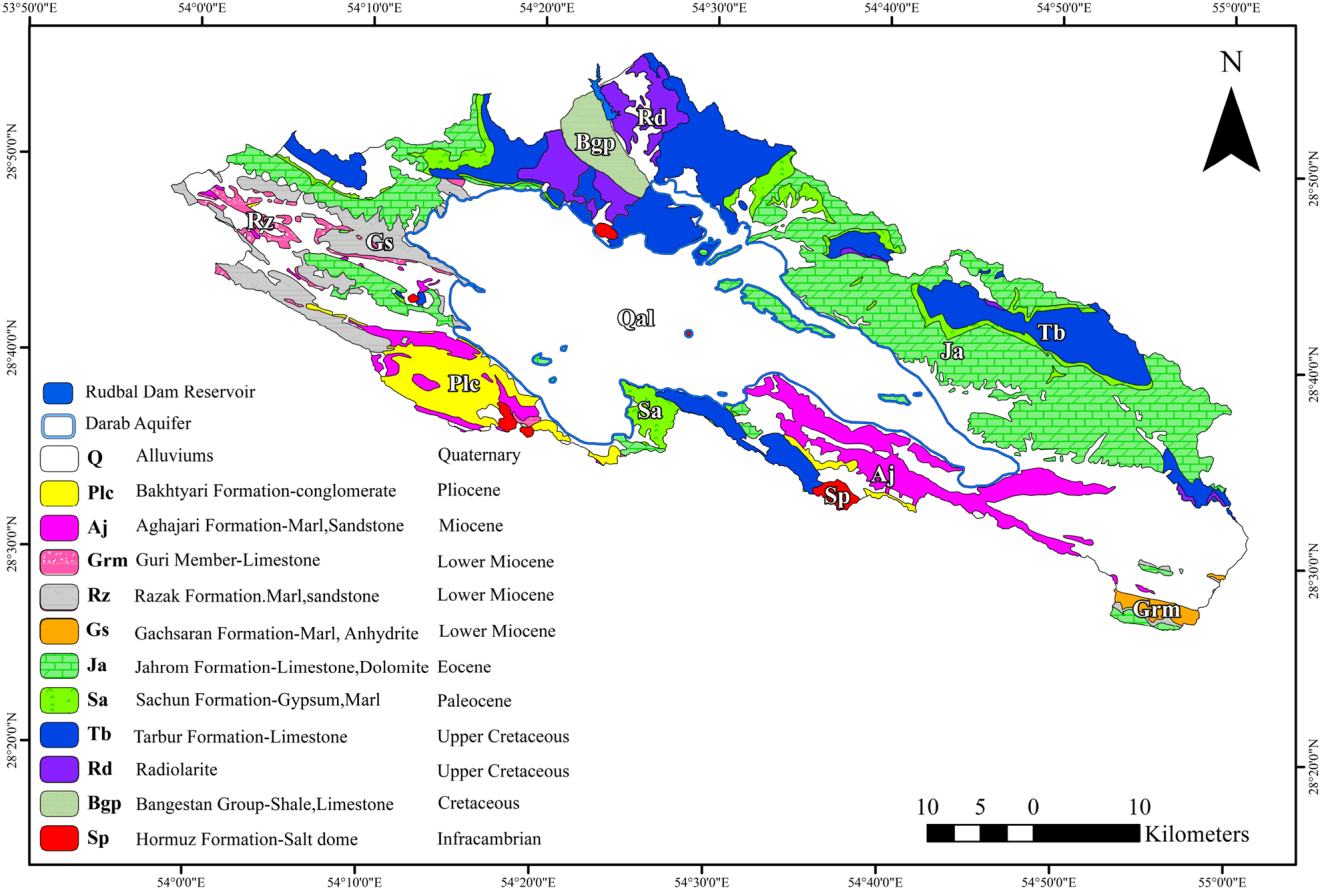
The map of the area study.

There are several geological formations outcrops in the study area, each defined as follows: (a) The Quaternary sedimentary , which constitutes a significant part of the Darab plain. (b) The Jahrom formation, comprising limestone layers. (c) The Gachsaran formation, located along the Champeh section, primarily composed of gypsum marl (Fig. 1).

## Methods and material

**Figure 2:**
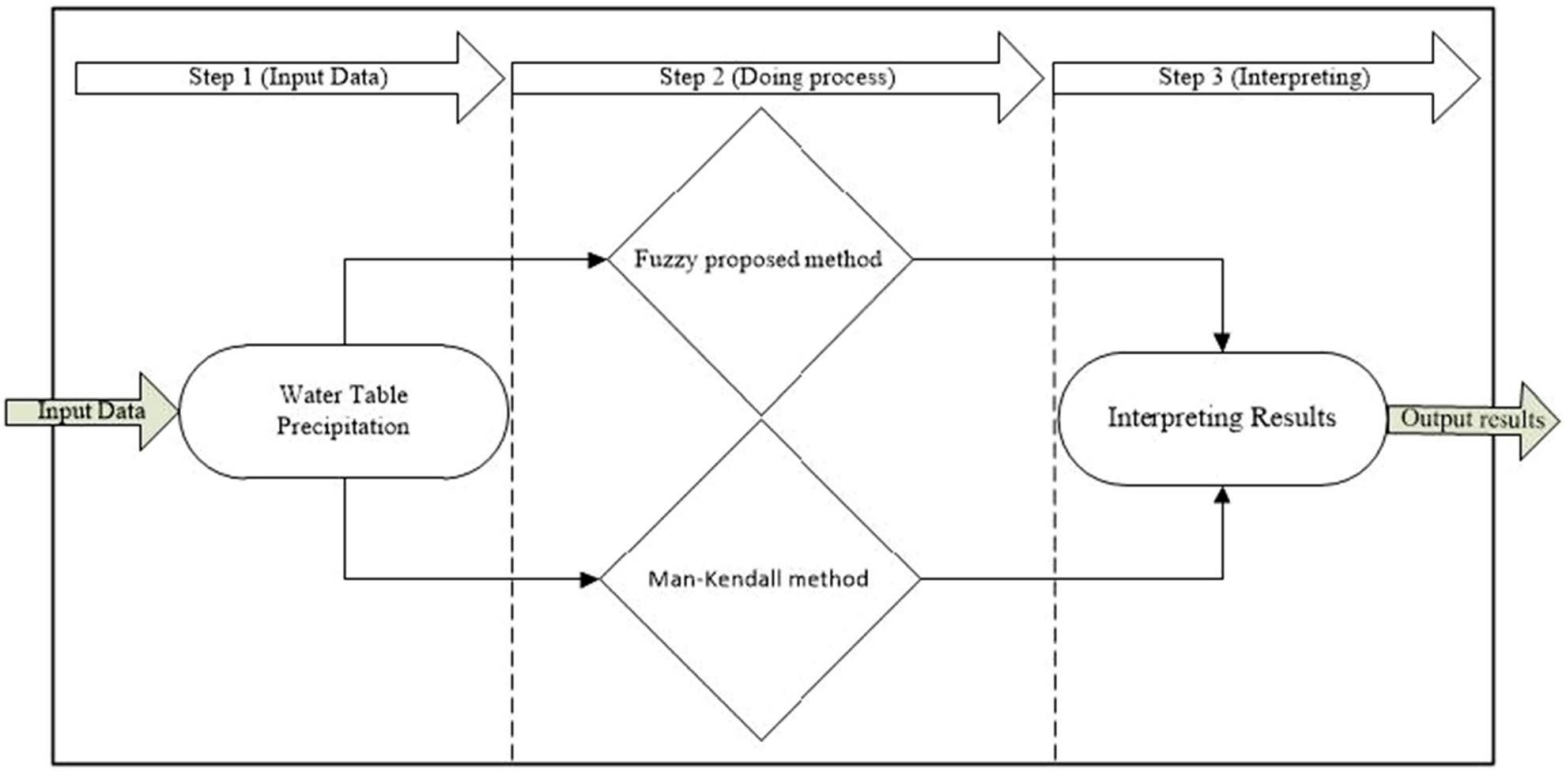
The steps of the study.

The paper proposes an innovative approach to evaluating the effects of the Dam on the Darab aquifer. The methodology consists of a series of well-defined steps, which are clearly illustrated in the accompanying Fig. 2.

### The input data

For this research, 19 observation well data were used for two steps. First, before building the dam from 2010 to 2015, and second, after building the dam from 2016 to 2021 (Table 1, Fig. 11). In addition, the precipitation data was also used for the proposed method in two steps: 2010 to 2015 and 2016 to 2021.

**Table 1:** Coordinates of piezometers.

Piezometers	X	Y
P1	258,111	3,185,213
P2	232,911	3,183,801
P3	261,176	3,183,331
P4	233,860	3,176,448
P5	245,402	3,178,083
P6	250,025	3,175,342
P7	253,216	3,177,361
P8	258,096	3,176,906
P9	266,425	3,175,396
P10	252,757	3,172,761
P11	258,646	3,172,604
P12	266,480	3,171,749
P13	245,799	3,168,612
P14	279,849	3,165,155
P15	238,639	3,184,105
P16	250,963	3,178,646
P17	253,323	3,179,927
P18	236,710	3,179,171
P19	240,359	3,169,555

### Mann–Kendall test (MK1/classical mann Kendall)

Mann–Kendall is a method for detecting trends in time series data. This method is a non-parametric approach that works even when the underlying distribution is unknown or non-normal.

The Mann–Kendall approach is defined as follows (Eq. 1)

1 $$\begin{aligned} S= & {} \sum \limits _{i=1}^{n-1}\sum \limits _{j=i+1}^{n}sgn(X_{j}-X_{i}) \end{aligned}$$

2 $$\begin{aligned} sng= & {} \left\{ \begin{array}{@{}ll@{}} 1, &{} \text {for x} > \text {0}\\ 0, &{} \text {for x = 0}\\ -1, &{} \text {for x} < \text {0} \end{array}\right. \end{aligned}$$

3 $$\begin{aligned} E(s)= & {} 0 \end{aligned}$$

4 $$\begin{aligned} Var(s)= & {} \frac{n(n-1)(2n+s)}{18} \end{aligned}$$

5 $$\begin{aligned} Z_{MK}= & {} \left\{ \begin{array}{@{}ll@{}} \frac{s - 1\sqrt{Var(S)}}{}, &{} \text {if s} > \text {0}\\ 0, &{} \text {if s = 0}\\ \frac{S+1}{\sqrt{Var(S)}}, &{} \text {if s} < \text {0} \end{array}\right. \end{aligned}$$

### Mann–Kendall test with trend-free pre-whitening

For the first time, MK-TFPW is presented by Yue and Wang^13^. The MK-TFPW approach is an advanced version of the classical Mann–Kendall test. It uses a pre-processing stage that eliminates any serial or temporal autocorrelation from the time series data, paving the way for a more precise trend analysis. There are several steps involved in doing MK-TFPW. The first step is (1) pre-whiting, which involves pre-processing the time series data to eliminate the impact of temporal autocorrelation.

(2) For detecting trends, the Mann–Kendall test is applied to the pre-processed data.

(3) To determine the significance of a trend, the Mann–Kendall test statistic calculates its strength and direction.

The Mann–Kendall approach is defined as follows (Eq. 1)

6 $$\begin{aligned} r_{k}=\frac{\frac{1}{n-k}\sum \nolimits _{i=1}^{n-k}(X_{i}-{\bar{X}})(X_{i+k}-{\bar{X}})}{\frac{1}{n}\sum \nolimits _{i=1}^{n}(X_{i}-{\bar{X}})^{2}} \end{aligned}$$

### Proposed fuzzy model

To determine similarities and differences in the behavior of the water table during precipitation, a combination of the standardization process, Fuzzy Inference System, and RMSE calculations are employed.

**Figure 3:**
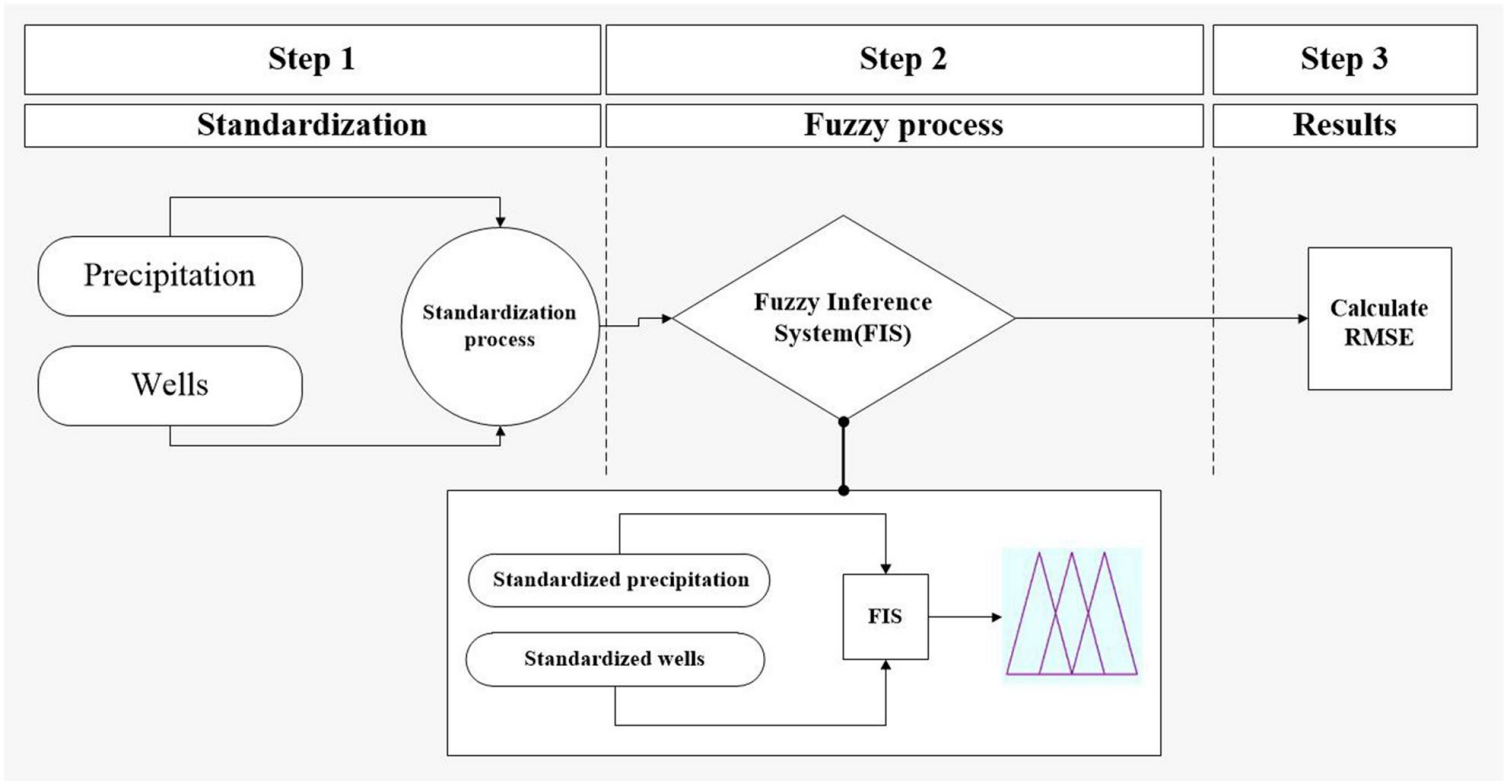
A brief explanation of the applied method.

Following is a brief explanation of the applied methods (Fig. 3):

#### Step1 (standardization)

Standardization is an important process for ensuring a high level of accuracy, reliability, and comparability in data. Furthermore, the application of standardization in groundwater studies considers various perspectives to achieve optimal outcomes, taking into account diverse factors, such as geological formations and human activities^17^.

It is important for consistency in data collection and analysis to utilize the Standardization process. Shown in the Tables 2, 3 is a summary of precipitation occurrences and 19 piezometric data for the Drab aquifer from 2000 to 2023.

**Table 2:** Precipitation and piezometers data (P1–P9).

Year	Month	Precipitation	P1	P2	P3	P4	P5	P6	P7	P8	P9
2000	1	36	1057.659	1057.786	1074.383	1047.688	1041.168	1048.637	1047.486	1068.235	1017.175
2000	2	0.6	1057.508	1057.852	1073.457	1047.704	1041.064	1047.713	1047.54	1067.976	1016.244
2000	3	0	1056	1059	1086	1044	1044	1040	1049	1044	1040
”	”	”	”	”	”	”	”	”	”	”	”
2023	3	0	1057.409	1058.191	1073.194	1047.609	1045.533	1048.766	1047.596	1068.869	1016.169

**Table 3:** Precipitation and piezometers data (P10–P19).

Year	Month	Precipitation	P10	P11	P12	P13	P14	P15	P16	P17	P18	P19
2000	1	36	1057.039	1040.007	1080.858	1001.062	1082.876	1057.233	1053.513	1048.142	1033.756	1030.049
2000	2	0.6	1057.07	1039.714	1080.895	1001.198	1082.704	1057.139	1053.702	1048.422	1034.764	1030.079
2000	3	0	1049	1050	1024	1036	1079	1040	1020	1048.945	1036	1030.935
”	”	”	”	”	”	”	”	”	”	”	”	”
2023	3	0	1057.289	1039.637	1080.967	1000.71	1082.636	1057.251	1053.591	1048.945	1034.733	1030.935

To standardize, the equation has been employed to scale the amount of precipitation and water table level within the range of 0–1. The formula is provided (Eq. 7)

7 $$\begin{aligned} S = \frac{X_{i}*1}{X_{max}} \end{aligned}$$

$S$: The value of the standardization. $X_i$: The specific data value you are interested in. $X_{\text {max}}$: The maximum data value in the dataset.

The formula essentially expresses $X_i$ as a value of the maximum value $X_{\text {max}}$.

#### Step 2 (fuzzy process)

Fuzzy logic is an influential approach that finds application across various scientific and technological fields by utilizing the qualitative aspects of expert knowledge^15^. Therefore, Fuzzy logic is a powerful tool that effectively deals uncertainty by collecting expert knowledge^16^. To conduct a comprehensive survey, one must thoroughly analyze fuzzy logic. First and foremost, fuzzy inference systems consist of four elements: fuzzification, fuzzy rule base, fuzzy inference and denazification. Fuzzification is a crucial process that transforms precise inputs into fuzzy sets. The fuzzy rule base consists of statements that establish relationships between fuzzy sets. Fuzzy inference is the process which applying fuzzy rules to make inferences about fuzzy sets. Denazification is the process of transforming indistinct outcomes into clear and precise outputs.

**Figure 4:**
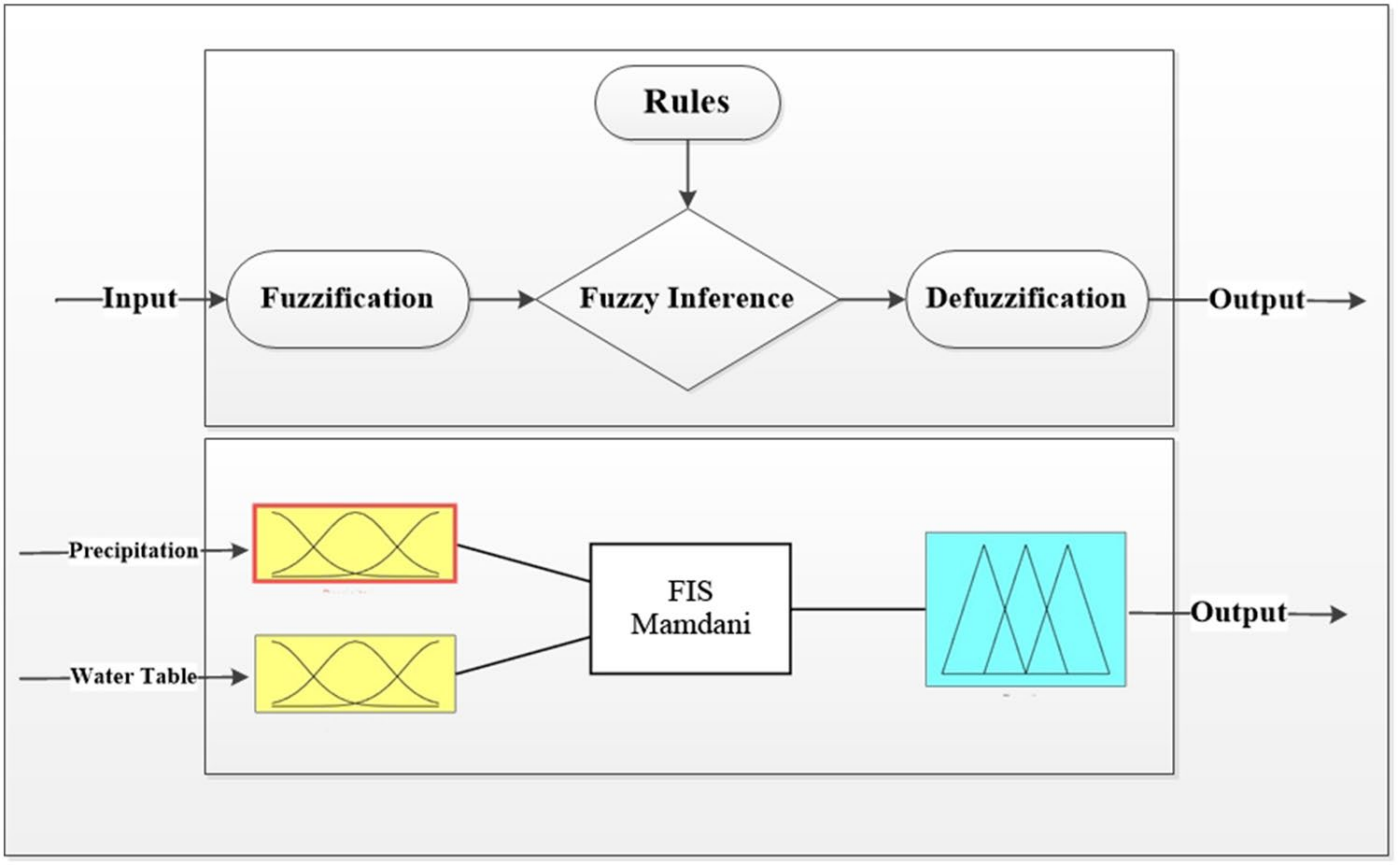
The steps of the fuzzy method.

Additionally, the Mamdani FIS is a fuzzy inference system used to investigate this study. Mamdani Fis is widely chosen by researchers in the field of water quality assessment due to its remarkable flexibility^18^ (Fig. 4).

**Fuzzification of the input and output parameters**

**Figure 5:**
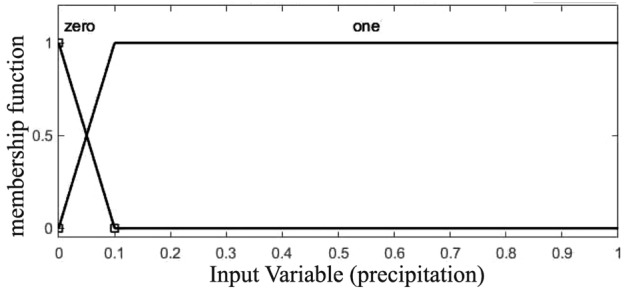
The shape of the input membership function (precipitation).

**Figure 6:**
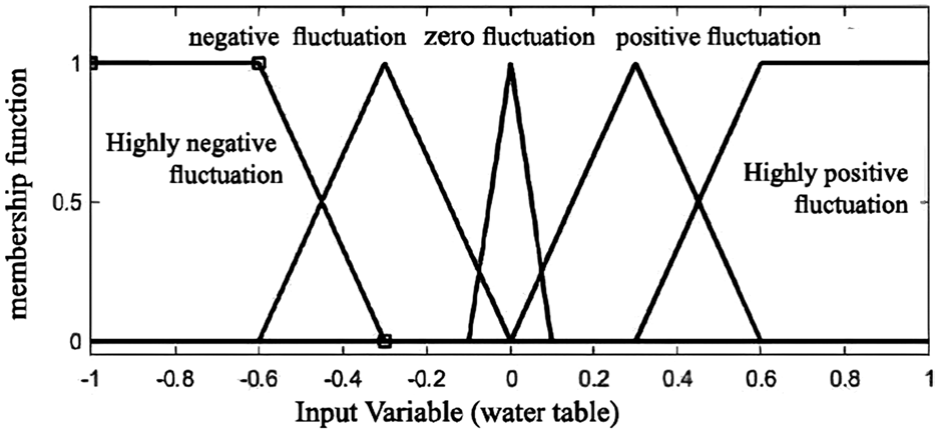
The shape of the input membership function (Water table).

**Figure 7:**
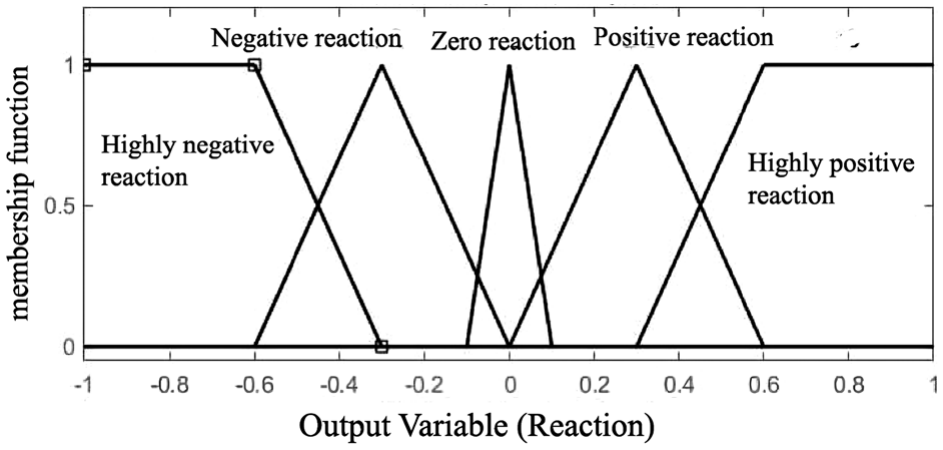
The shape of the output membership function (Reaction).

One of the key steps in fuzzy logic is the process of fuzzifying input data, wherein precise input values are transformed into fuzzy sets. Furthermore, the effectiveness of the Fuzzy Inference System (FIS) is notable, especially concerning the determination of the number of membership functions for all inputs^18^. Add to this, membership functions rely on the subject which is to be solved^19^. Trapezoidal and triangular membership functions are selected as the best choices, due to their appropriateness for representing the data’s characteristics^18^. Overall,The process involves converting specific input values, such as observed water table levels from piezometers and precipitation data, into fuzzy sets using predetermined membership functions. This helps to address uncertainty and imprecision in the measurements by transforming exact values into fuzzy sets. The rules and the shape of the membership function have been adjusted with the goal of achieving an optimal fuzzy system (Figs. 5, 6 and 7).

#### Step 3 (calculating RMSE)

There are a few indicators to check performance of the results.Two common performance indicators are Root Mean Square Error (RMSE) and Coefficient of Determination (R2)^20^.

To evaluate the performance of the fuzzy logic model, root mean square error (RMSE) and the coefficient of determination (R2) have been chosen. This indicator is shown by Eq. (8).

8 $$\begin{aligned} RMSE = \sqrt{\frac{1}{n}\sum _{i=1}^{n}(Y_i - \hat{Y}_i)^2} \end{aligned}$$

### Results and discursion

**Figure 8:**
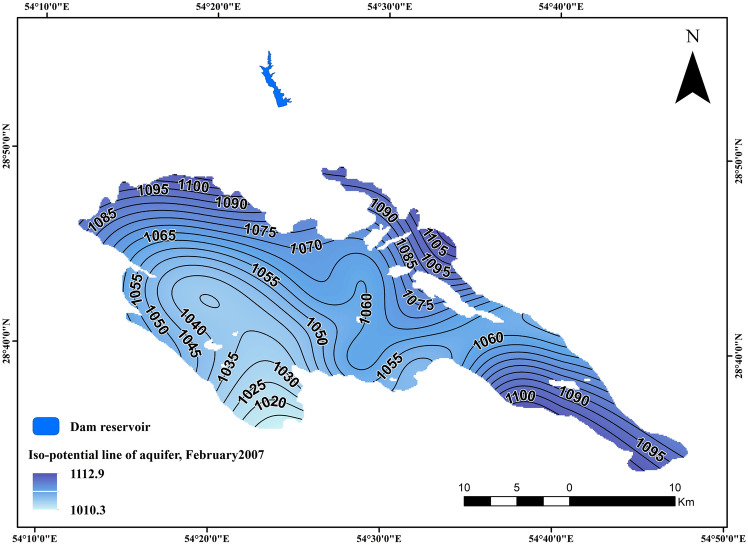
The iso-potential maps for the Darab aquifer at different time (2010).

**Figure 9:**
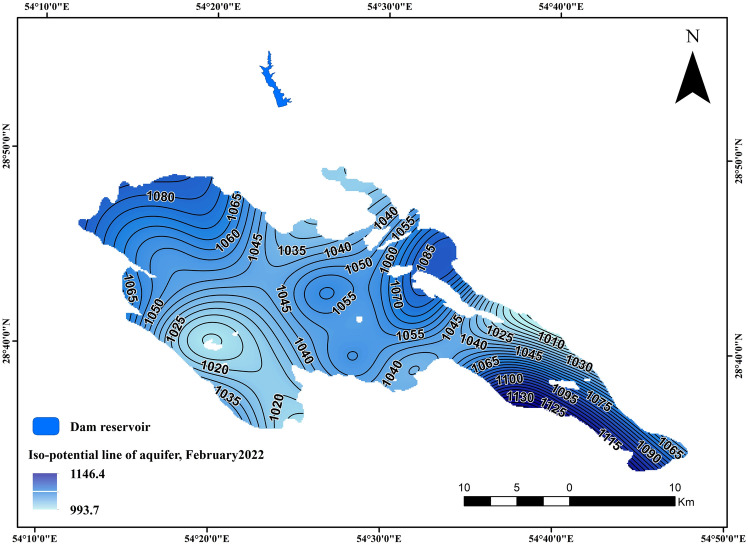
The iso-potential maps for the Darab aquifer at different time (2022).

Shown in the pictures are the iso-potential maps for the Darab aquifer at different times (i.e., February 2010 and 2022). Overall, the main flow direction is from north and northwest to south and southeast. Additionally, the presence of the Darab dam is another factor to consider in 2022 (Figs. 8, 9).

#### Result of the purposed method

**Figure 10:**
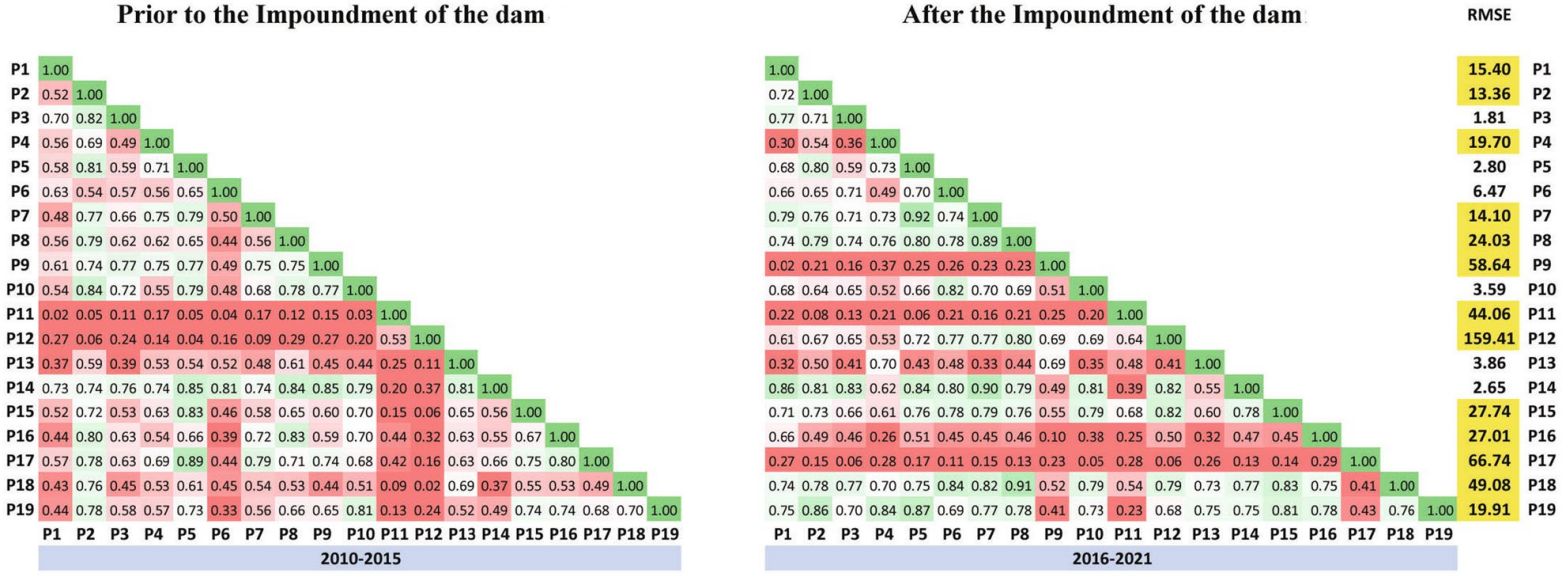
The amount of the correlation between piezometers for two different time (from 2010 to 2015 and from 2016 to 2021).

The results of the proposed method were tracked for two time periods: from 2010 to 2015 and from 2016 to 2021, both before and after the dam dewatering and the amount of the correlation between piezometers with each other, as depicted in the Fig. 10.

All in all, it can be inferred from the data that the degree of correlation between before and after dam dewatering has undergone significant changes in certain piezometers. Observing the RMSE, there is a clear indication that the behavior of the piezometers (i.e., P1, P9, P11, P12, P15, P16, P17, P18, and P19) undergoes significant changes after the dam dewatering.

#### Result of the Mann–Kendall test

Table 4 presents the results of the Mann–Kendall test for 19 piezometers. Overall, it is evident that Kendall’s tau, p-value, and Sen’s slope have been analyzed.

Additionally, if the p-value is below 5%, no significant trend is detected in the time series. Conversely, Kendall’s tau and Sen’s slope indicate the strength and direction of monotonic trends as well as the slope of the observed trend in the data.

It is clear that thirteen out of the eighteen piezometers exhibit a noticeable trend (i.e., P1, P2, P4, P5, P9, P10,P11, P12, p15, P16, p17, P18 and P19). Among these, five of the thirteen piezometers show an increasing trend. However, the table also suggests that six piezometers show no significant trend(I.e.,P3,P6,P7,P8,P13 and P14).

**Table 4:** Summary of data.

Well	Kendall’s tau	p-value	Sen’s slope	Trend
P1	− 0.417	0.001	− 0.043	Decreasing
P2	− 0.206	0.011	− 0.021	Decreasing
P3	0.083	0.539	0.017	No trend
P4	− 0.600	$< 0.0001$	− 0.041	Decreasing
P5	− 0.772	$< 0.0001$	− 0.034	Decreasing
P6	0.051	0.413	0.001	No trend
P7	0.156	0.126	0.010	No trend
P8	0.072	0.236	0.003	No trend
P9	− 0.355	0.007	− 0.067	Decreasing
P10	− 0.837	$< 0.0001$	− 0.037	Decreasing
P11	− 0.892	$< 0.0001$	− 0.074	Decreasing
P12	− 0.569	$< 0.0001$	− 0.019	Decreasing
P13	− 0.039	0.655	− 0.022	No trend
P14	0.049	0.700	0.005	No trend
P15	0.380	0.006	0.046	Increasing
P16	0.275	$< 0.0001$	0.036	Increasing
P17	0.540	$< 0.0001$	0.051	Increasing
P18	0.349	0.017	0.032	Increasing
P19	0.310	0.010	0.026	Increasing

#### Interpreting results

Table 5 presents the final results of the two investigated methods, namely the Fuzzy Purpose Method and the Mann–Kendall Method.

**Figure 11:**
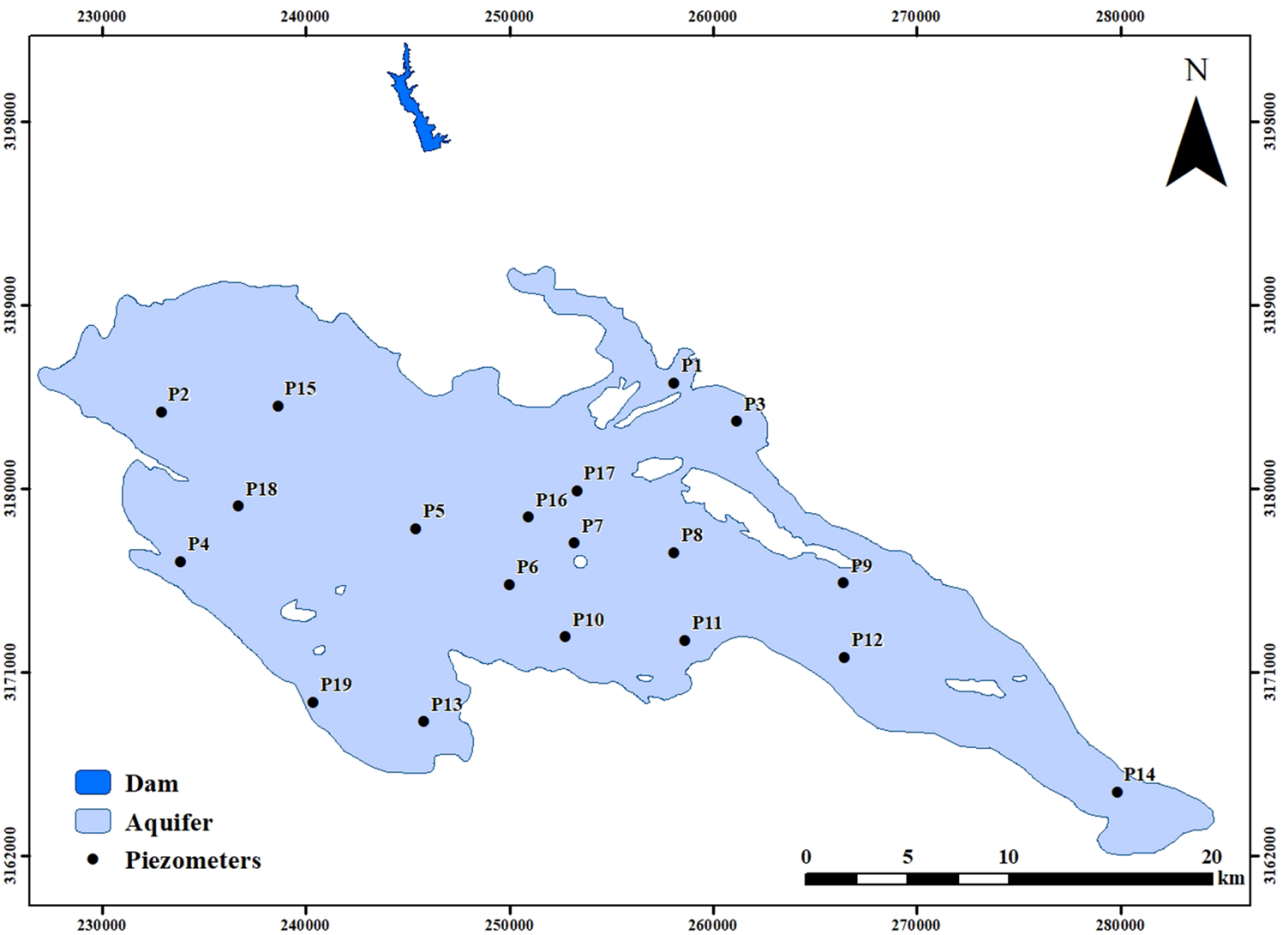
The location of the piezometers.

In general, it is evident that 70% of the results from both methods exhibit similarity. However, differences between the two methods arise due to variations in the calculated functions and input data. Based on the data, it is apparent that the outcomes generated by the Fuzzy Purpose Method align better with reality compared to the Mann–Kendall Method. Specifically, the Mann–Kendall Method suggests that certain variables, such as P3, P7, and P8, do not show any discernible trends following the dam impoundment (Fig. 11). In contrast, the Fuzzy Purpose Method provides more accurate results, which diverge from those obtained using the Mann–Kendall Method.

**Table 5:** Summary of well data.

Piezometers	Kendall’s tau	Trend	RMSE of purposed method
P1	− 0.417	**Decreasing**	**15.40**
P2	− 0.206	**Decreasing**	**13.36**
P3	0.083	No trend	1.81
P4	− 0.600	**Decreasing**	**19.70**
P5	− 0.772	**Decreasing**	2.80
P6	0.051	No trend	6.47
P7	0.156	No trend	**14.10**
P8	0.072	No trend	**24.03**
P9	− 0.355	**Decreasing**	**58.64**
P10	− 0.837	**Decreasing**	3.59
P11	− 0.892	**Decreasing**	** 44.06**
P12	− 0.569	**Decreasing**	**159.41**
P13	− 0.039	No trend	3.86
P14	0.049	No trend	2.65
P15	0.380	**Increasing**	**27.74**
P16	0.275	**Increasing**	**27.01**
P17	0.540	**Increasing**	**66.74**
P18	0.349	** Increasing**	**49.08**
P19	0.310	**Increasing**	**19.91**

Significant values are given in bold.

**Figure 12:**
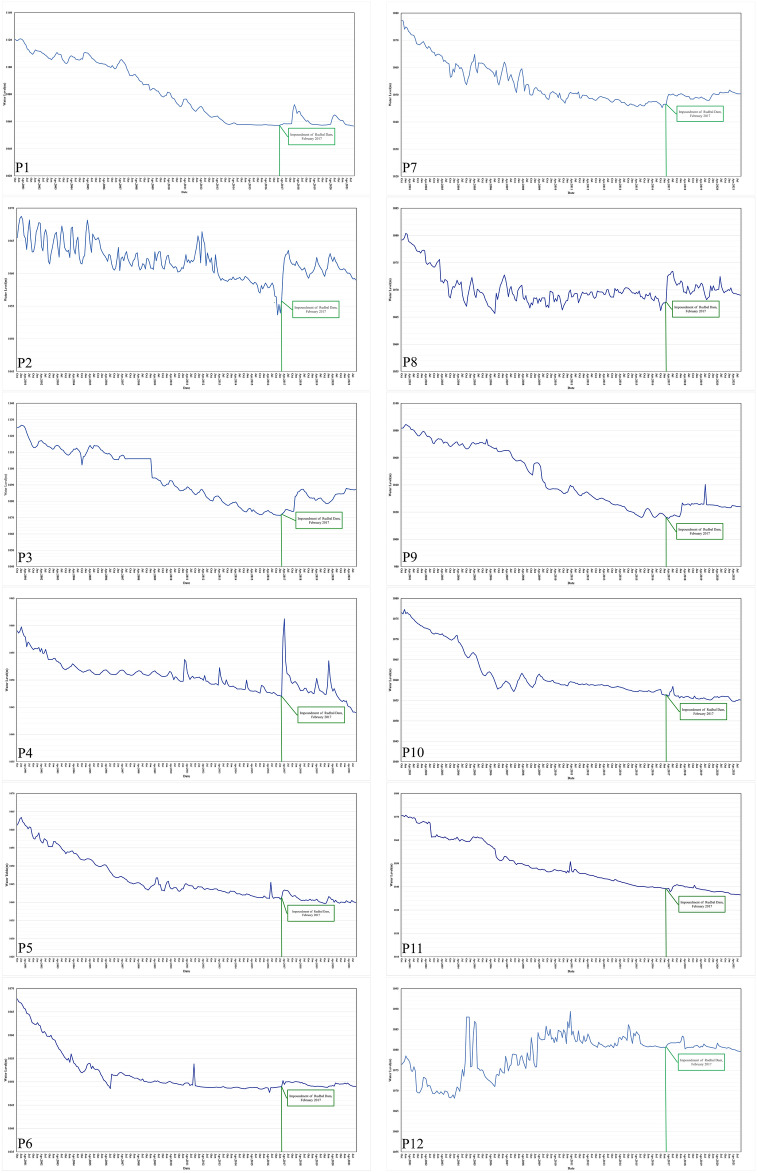

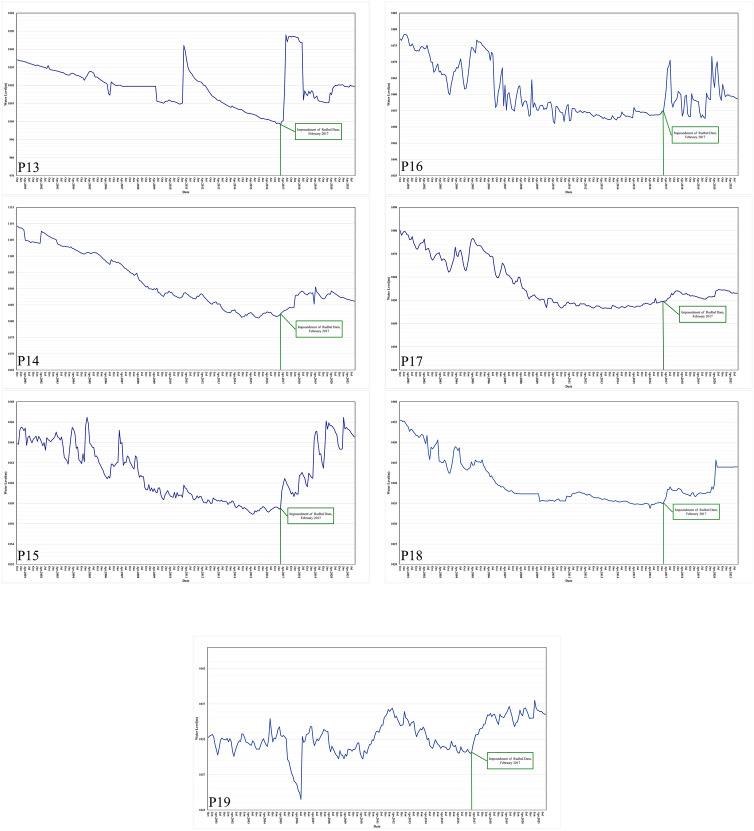
Trend of the piezometers.

## Conclusion

It is always important to investigate the effect of the presence of a dam on groundwater management. Therefore, one of these effects can be the change in the water level of some piezometers, caused by the injection of water from the dam’s reservoir into the geological set. In this study, for the first time, the behavior of the piezometers was surveyed before and after the dam’s construction.

The effect of a dam can be investigated using a variety of methods, but this study utilized a new fuzzy method for investigating the impact of Robal Dam on the Drab aquifer (i.e., the proposed method), and then applied the Man–Kendal statistical method to interpret the obtained results of the new method. Add to this, By employing a combination of the Fuzzy Inference System (FIS) and Root Mean Square Error (RMSE) calculations, the research provides a robust analysis of water table behavior during precipitation events. The findings highlight the efficacy of the Fuzzy Purpose Method over the Mann–Kendall Method, showing a 70% similarity in outcomes but with better alignment to real-world data when using fuzzy logic. Nineteen piezometers were investigated in the Darab aquifer for two different periods. (i.e., 2010–2015 and 2016–2021). The water table behavior is shown in Figs. 8 and 9 (i.e., before and after the construction of the Rodbal dam ).

First, 70% of the piezometer behavior changed after the construction of the Rodbal dam (Table 5). On the other hand, the results of the fuzzy proposed method show more of the changed piezometer, which is not in the results of the Mann–Kendall methods. On the one hand, the change’s result and amount are illustrated in Fig. 12.

The significance of these findings lies in the ability of the Fuzzy Purpose Method to more accurately reflect the complexities of groundwater systems influenced by dam construction. Finally, it appears that the fuzzy proposed method works in practice and can reveal details of changes. However, there are many limitations to obtaining the best results, which leaves room for future research.

## Data Availability

The datasets used and/or analyzed during the current study available from the corresponding author on reasonable request.
